# Resistance to the mTOR-inhibitor RAD001 elevates integrin *α*2- and *β*1-triggered motility, migration and invasion of prostate cancer cells

**DOI:** 10.1038/bjc.2012.313

**Published:** 2012-07-10

**Authors:** I Tsaur, J Makarević, E Juengel, M Gasser, A-M Waaga-Gasser, M Kurosch, M Reiter, S Wedel, G Bartsch, A Haferkamp, C Wiesner, R A Blaheta

**Affiliations:** 1 Department of Urology, Goethe-University, Frankfurt am Main 60590, Germany; 2 Department of Surgery I, University of Wuerzburg, Wuerzburg 97080, Germany

**Keywords:** prostate cancer, RAD001 resistance, integrins, Akt, migration

## Abstract

**Background:**

Inhibitors of the mammalian target of rapamycin (mTOR) might become a novel tool to treat advanced prostate cancer. However, chronic drug exposure may trigger resistance, limiting the utility of mTOR inhibitors.

**Methods:**

Metastatic potential of PC3 prostate cancer cells, susceptible (PC3^par^) or resistant (PC3^res^) to the mTOR-inhibitor RAD001 was investigated. Adhesion to vascular endothelium or immobilised collagen, fibronectin and laminin was quantified. Motility, migration and invasion were explored by modified Boyden chamber assay. Integrin *α* and *β* subtypes were analysed by flow cytometry, western blotting and real-time PCR. Integrin-related signalling, EGFr, Akt, p70S6kinase and ERK1/2 activation were determined.

**Results:**

Adhesion was reduced, whereas motility, migration and invasion were enhanced in PC3^res^. The *α*2 and *β*1 integrin subtypes were dramatically elevated, integrins *α*1 and *α*6 were lowered, whereas *α*5 was nearly lost in PC3^res^. Activation of the Akt signalling pathway was strongly upregulated in these cells. Treating PC3^par^ cells with RAD001 reduced motility, migration and invasion and deactivated Akt signalling. Blocking studies revealed that *α*2 and *β*1 integrins significantly trigger the motile behaviour of the tumour cells.

**Conclusion:**

Chronic RAD001 treatment caused resistance development characterised by distinct modification of the integrin-expression profile, driving prostate cancer cells towards high motility.

Prostate cancer, once metastasised, is difficult to treat. Surgical castration or hormonal manipulation provides initial success. However, patients progressively become hormone-resistant. During the last years, specific target proteins have been identified, which are involved in neoplastic development and tumour progression. *In vitro* investigation points to a close relationship between hormone resistance and activation of the mammalian target of rapamycin (mTOR) pathway (Wu *et al*, 2010). Analysis of tumour specimens has documented the association between mTOR variations and prostate cancer risk (Campa *et al*, 2011). Indeed, most patients with prostate cancer have at least one activated component of the mTOR signalling pathway (Kremer *et al*, 2006; Dai *et al*, 2009).

Hence, inactivating mTOR could become an attractive option to treat advanced prostate cancer. Among the number of mTOR inhibitors that have have been developed, the rapamycin analogues temsirolimus and RAD001 (everolimus) are the most prevalent in clinical use. Both have received US Food and Drug Administration approval, which however, is restricted to the treatment of advanced renal cell carcinoma. The relevance of temsirolimus and RAD001 in treating prostate cancer is still unclear. Although preclinical studies show mTOR inhibitors reverting prostatic neoplasia and reducing cell growth and proliferation (Morgan *et al*, 2009), the clinical experience of mTOR inhibition in men with castrate-resistant prostate cancer has been disappointing. Only a few patients have benefited from an mTOR inhibition-based regimen, and disease progression inevitably occured during treatment (Amato *et al*, 2008; Armstrong *et al*, 2010). It has, therefore, been argued that chronic drug exposure triggers the development of resistance, ultimately limiting the utility of mTOR inhibitors (Amato *et al*, 2008). Knowledge about the precise mechanism of resistance, however, is limited. Based on a RAD001-resistant prostate cancer cell line, we recently reported that drug non-responsiveness is characterised by an increased level of cdk1 and cyclin B, which counteracts growth-blocking effects of this drug (Tsaur *et al*, 2011). These studies have now been extended to explore the consequences of RAD001 resistance on the metastatic behaviour of prostate tumour cells. Additionally, the activity of RAD001-target proteins, as well as the expression pattern of *α* and *β* integrin adhesion receptors in resistant and non-resistant tumour cells, was analysed.

## Materials and methods

### Cell culture

The human prostate tumour cell line PC3 was obtained from DSMZ (Braunschweig, Germany). Tumour cells were grown and subcultured in RPMI 1640 (Gibco/Invitrogen, Karlsruhe, Germany) containing 10% fetal calf serum (FCS), 2% HEPES buffer (1 ℳ, pH 7.4), 2% glutamine and 1% penicillin/streptomycin. The RAD001-resistant subline was developed by 12 months of exposure to RAD001, starting at 1 nℳ and increasing stepwise to 1 *μ*ℳ. The control cells were designated PC3^par^, the resistant variant was termed PC3^res^.

Human endothelial cells (HUVECs) were isolated from human umbilical veins and harvested by enzymatic treatment with dispase (Gibco/Invitrogen). Human endothelial cells were grown in Medium 199 (M199; Biozol, Munich, Germany), supplemented with 10% FCS, 10% pooled human serum, 20 *μ*g ml^−1^ endothelial cell growth factor (Boehringer, Mannheim, Germany), 0.1% heparin, 100 ng ml^−1^ gentamycin and 20 mℳ HEPES buffer (pH 7.4). Subcultures from passages 2–6 were selected for experimental use.

### RAD001

RAD001 (provided by Novartis Pharma AG, Basel, Switzerland) was dissolved in DMSO as 10 mℳ stock solution and stored in aliquots at −20 °C. Prior to the experiments, RAD001 was diluted in cell culture medium. To analyse the influence of RAD001 on chemotactic movement, migration and invasion of PC3^par^ versus PC3^res^ cells, cell culture medium of PC3^res^ cells containing 1 *μ*ℳ RAD001 was replaced by RAD001-free medium to avoid unspecific effects. A medium change was also carried out in the PC3^par^ cell culture system. After 3 days, 5 nℳ RAD001 was added to both PC3^par^ versus PC3^res^ cells (controls were treated with fresh medium without RAD001) and chemotactic movement, migration and invasion were analysed.

To exclude toxic effects of the compound, cell viability was determined by trypan blue (Gibco/Invitrogen). For apoptosis detection, the expression of annexin V/propidium iodide (PI) was evaluated using the Annexin V-FITC Apoptosis Detection kit (BD Pharmingen, Heidelberg, Germany). Tumour cells were washed twice with PBS and then incubated with 5 *μ*l of Annexin V-FITC and 5 *μ*l of PI in the dark for 15 min at room temperature. Cells were analysed on a FACScalibur (BD Biosciences, Heidelberg, Germany). The percentage of apoptotic cells (early and late) in each quadrant was calculated using CellQuest software (BD Biosciences).

### Tumour cell adhesion

To analyse tumour cell adhesion, HUVECs were transferred to six-well multiplates (Falcon Primaria; BD Biosciences) in complete HUVEC medium. When confluency was reached, PC3^par^ or PC3^res^ cells were detached from the culture flasks by accutase treatment (PAA Laboratories, Cölbe, Germany) and 0.5 × 10^6^ cells were then added to the HUVEC monolayer for 30, 60 or 120 min. Subsequently, non-adherent tumour cells were washed off using warmed (37 °C) Medium 199. The remaining cells were fixed with 1% glutaraldehyde. Adherent tumour cells were counted in five different fields of a defined size (5 × 0.25 mm^2^) using a phase contrast microscope and the mean cellular adhesion rate was calculated.

### Attachment to extracellular matrix components

Six-well plates were coated with collagen G (extracted from calfskin, consisting of 90% collagen type I and 10% collagen type III; seromed; diluted to 400 *μ*g ml^−1^ in PBS), laminin (derived from the Engelbreth–Holm–Swarm mouse tumour; diluted to 50 *μ*g ml^−1^ in PBS; BD Biosciences) or fibronectin (derived from human plasma; diluted to 50 *μ*g ml^−1^ in PBS; BD Biosciences) overnight. Unspecific cell binding was evaluated by culture plates treated with poly-𝒟-lysine (Nunc, Wiesbaden, Germany). Plastic dishes were served as the background control. Plates were washed with 1% bovine serum albumin (BSA) in PBS to block nonspecific cell adhesion. Thereafter, 0.5 × 10^6^ tumour cells were added to each well for 60 min. Subsequently, non-adherent tumour cells were washed off, the remaining adherent cells were fixed with 1% glutaraldehyde and counted microscopically. The mean cellular adhesion rate, defined by adherent cells_coated well_−adherent cells_background_, was calculated from five different observation fields.

### Measurement of tumour cell motility (chemotaxis), migration and invasion

Serum-induced chemotactic movement was examined using six-well Transwell chambers (Greiner, Frickenhausen, Germany) with 8-*μ*m pores. A total of 0.5 × 10^6^ PC3^par^ versus PC3^res^ cells per ml were placed in the upper chamber in serum-free medium. To evaluate cell migration, Transwell chambers were precoated with collagen (400 *μ*g ml^−1^). Cell invasion was explored by coating the Transwell chambers with collagen (400 *μ*g ml^−1^), which were then overlaid with HUVEC. The lower chamber contained 10% serum. After 20 h incubation, the upper surface of the Transwell membrane was gently wiped with a cotton swab to remove non-migrating cells. Cells, which had moved to the lower surface of the membrane, were stained using hematoxylin and counted microscopically. The mean chemotaxis, migration or invasion rate was calculated from five different observation fields.

### Integrin surface expression

PC3^par^ versus PC3^res^ cells were washed in blocking solution (PBS, 0.5% BSA) and then incubated for 60 min at 4 °C with phycoerythrin (PE)-conjugated monoclonal antibodies directed against the following integrin subtypes: anti-*α*1 (IgG1; clone SR84, dilution 1 : 1000), anti-*α*2 (IgG2a; clone 12F1-H6, dilution 1 : 250), anti-*α*3 (IgG1; clone C3II.1, dilution 1 : 1000), anti-*α*4 (IgG1; clone 9F10, dilution 1 : 200), anti-*α*5 (IgG1; clone IIA1, dilution 1 : 5000), anti-*α*6 (IgG2a; clone GoH3, dilution 1 : 200), anti-*β*1 (IgG1; clone MAR4, dilution 1 : 2500), anti-*β*3 (IgG1; clone VI-PL2, dilution 1 : 2500) or anti-*β*4 (IgG2a; clone 439–9B, dilution 1 : 250; all: BD Biosciences). Integrin expression of tumour cells was then measured using a FACscan (BD Biosciences; FL-2H (log) channel histogram analysis; 1 × 10^4^ cells per scan) and expressed as mean fluorescence units. A mouse IgG1-PE (MOPC-21) or IgG2a-PE (G155–178; all: BD Biosciences) was used as an isotype control.

### Western blot analysis

To explore the integrin protein level after 24 h drug incubation, tumour cell lysates were applied to a 7% polyacrylamide gel and electrophoresed for 90 min at 100 V. The protein was then transferred to nitrocellulose membranes. After blocking with non-fat dry milk for 1 h, the membranes were incubated overnight with the monoclonal antibodies listed above. Additionally, integrin-related signalling was explored by anti-integrin-linked kinase (ILK; clone 3, dilution 1 : 1000), anti-focal adhesion kinase (FAK; clone 77, dilution 1 : 1000) and anti-phospho-specific FAK (pY397; clone 18, dilution 1 : 1000) antibodies (all: BD Biosciences). HRP-conjugated goat-anti-mouse IgG (Upstate Biotechnology, Lake Placid, NY, USA; dilution 1 : 5.000) served as the secondary antibody. The membranes were briefly incubated with ECL detection reagent (ECL, Amersham/GE Healthcare, München, Germany) to visualise the proteins and then analysed by the Fusion FX7 system (Peqlab, Erlangen, Germany). *β*-actin (1 : 1.000; Sigma, Taufenkirchen, Germany) served as the internal control.

### Real-time (RT)–qPCR

RT–qPCR was also done in triplicate. cDNA synthesis was performed using 3 *μ*g of total RNA per sample according to the manufacturer’s protocol by AffinityScript QPCR cDNA Synthesis Kit (Stratagene, Amsterdam, The Netherlands). Quantitative gene-expression analysis by RT–PCR was performed by the Mx3005p (Stratagene) using SYBR-Green SuperArray (SABioscience Corporation, Valencia, CA, USA) and SuperArray primer sets: GAPDH (NM_002046.3, Hs.592355), integrin *α*1 (ITGA1, NM_181501, Hs.644352), integrin *α*2 (ITGA2, NM_002203, Hs.482077), integrin *α*3 (ITGA3, NM_002204, Hs.265829), integrin *α*4 (ITGA4, NM_000885, Hs.694732), integrin *α*5 (ITGA5, NM_002205, Hs.505654), integrin *α*6 (ITGA6, NM_000210, Hs.133397), integrin *β*1 (ITGB1, NM_002211, Hs.643813), integrin *β*3 (ITGB3, NM_000212, Hs.218040) and integrin *β*4 (ITGB4, NM_000213, Hs.632226; all: SABioscience Corporation). Calculation of the relative expression of each gene was done by the ΔΔCt method in the analysis programme of SABioscience Corporation. The housekeeping gene GAPDH was used for normalisation.

### Cell signalling

Cell signalling was explored by using the following monoclonal antibodies: Akt (IgG1, clone 55, dilution 1 : 500), phospho Akt (pAkt; IgG1, clone 104A282, dilution 1 : 500), EGFr (IgG1, clone 13/EGFR, dilution 1 : 500), phospho EGFr (pEGFr; IgG1, clone 74, dilution 1 : 1000), ERK1 (IgG1, clone MK12, dilution 1 : 5000), ERK2 (IgG2b, clone 33, dilution 1 : 5000), phospho ERK1/2 (pERK; IgG1, clone 20A, dilution 1 : 1000; all: BD Biosciences), p70S6k (IgG, clone 49D7, dilution 1 : 1000) and phospho p70S6k (pp70S6k; IgG, clone 108D2, dilution 1 : 1000; all: New England Biolabs, Frankfurt, Germany).

### Blocking studies

PC3^par^ and PC3^res^ cells were preincubated for 60 min with function-blocking anti-integrin *β*1 (clone 6S6), anti-integrin *α*2 (clone P1E6) or anti-integrin *α*5 (clone P1D6; all: Millipore, Schwalbach, Germany) monoclonal antibodies (20 *μ*g ml^−1^). Controls remained untreated. Cells were then subjected to the chemotaxis and migration assay as indicated above. Adhesion to immobilised collagen was evaluated additionally. An anti-Akt function-blocking antibody was used to analyse the influence of Akt on PC3^par^ and PC3^res^ cell chemotaxis (Akt inhibitor VIII, 20 *μ*g ml^−1^; Chemdea, Ridgewood, NJ, USA).

### Statistics

All experiments were performed three to six times. Statistical significance was investigated by the Wilcoxon Mann–Whitney *U*-test. Differences were considered statistically significant at a *P*-value <0.05.

## Results

### Adhesion characteristics

**Figure 1:**
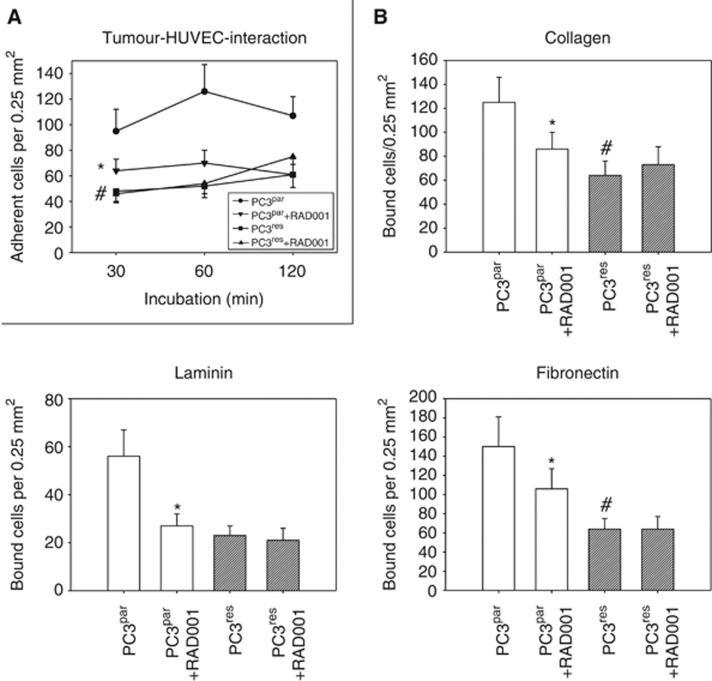
Cell–cell and cell–matrix interaction of PC3^par^ versus PC3^res^ cells. (**A**) Adhesion to HUVEC. PC3^par^ and PC3^res^ cells were treated with fresh medium (without RAD001) for 3 days and then added to HUVEC monolayers for 30, 60 or 120 min. (**B**) Adhesion to extracellular matrix proteins. PC3^par^ and PC3^res^ cells were treated with fresh medium (without RAD001) for 3 days and then added to immobilised collagen, laminin or fibronectin for 60 min. The adhesion rate of PC3^par^ versus PC3^res^ cells was compared in both experimental settings. Adhesion rate of PC3^par^ and PC3^res^ cells was also compared with the number of PC3^par^ and PC3^res^ cells treated with fresh medium for 3 days and subsequently with 5 nℳ RAD001. Mean values were calculated from five counts. Mean adhesion (**A**) or binding capacity (**B**) is depicted as adherent cells per 0.25 mm^2^. One representative of six experiments is shown. *indicates significant difference between the PC3 subline not treated with 5 nℳ RAD001 and the PC3 subline treated with 5 nℳ RAD001. ^#^indicates significant difference between PC3^par^ and PC3^res^ cells.

Dynamic evaluation of tumour cell–endothelial cell interaction revealed that more PC3^par^ cells adhered to HUVEC over time than did PC3^res^ cells (Figure 1A). Addition of 5 nℳ RAD001 significantly reduced the adhesion capacity of PC3^par^ but not of PC3^res^ cells. A similar phenomenon was seen in the extracellular matrix binding assay. More PC3^par^ cells bound to immobilised collagen, laminin or fibronectin than PC3^res^ cells (Figure 1B), and application of 5 nℳ RAD001 resulted in a diminished attachment rate of PC3^par^ but not of PC3^res^ cells.

### Tumour motility, migration and invasion

**Figure 2:**
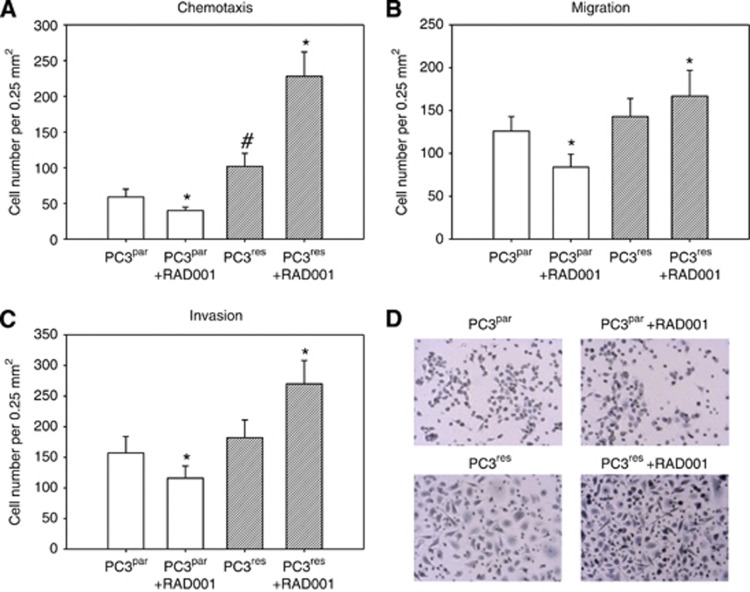
RAD001 resistance alters PC3 chemotaxis (**A, D**), migration (**B**) and invasion (**C**) as assessed in a Transwell chamber assay. PC3^par^ and PC3^res^ cells were used, as well as PC3^par^ and PC3^res^ cells, additionally treated with 5 nℳ RAD001, as indicated in Materials and Methods. To evaluate chemotaxis, tumour cells were seeded in the upper chamber in serum-free medium, and 10% FCS, as the chemoattractant, was placed in the lower well. To evaluate cell migration, Transwell chambers were precoated with collagen. Invasion was analysed by adding the tumour cells to the upper chamber, which was coated with collagen and overlaid with HUVEC. Cells, which moved to the lower surface of the membrane, were stained using hematoxylin and counted. (**D**) Representative chemotaxis assays. Mean values were calculated from five counts and depicted as cell number per 0.25 mm^2^. One representative of six experiments is shown. *indicates significant difference between the PC3 subline not treated with 5 nℳ RAD001 and the PC3 subline treated with 5 nℳ RAD001. ^#^indicates significant difference between PC3^par^ and PC3^res^ cells.

Chemotactic movement was significantly elevated in PC3^res^, compared with PC3^par^ cells (Figure 2A). Furthermore, application of 5 nℳ RAD001 blocked chemotaxis of PC3^par^ but strongly increased the motile activity of PC3^res^ cells. PC3^res^ cells also tended to cross collagen (Figure 2B) or HUVEC (Figure 2C) at a higher rate than PC3^par^ cells did. The addition of 5 nℳ RAD001 decreased migration and invasion of PC3^par^ cells. In contrast, migration and invasion of PC3^res^ cells were upregulated in the presence of 5 nℳ RAD001. Figure 2D shows chemotaxis of penetrated PC3^par^ cells, PC3^par^ cells treated with RAD001, PC3^res^ cells and PC3^res^ cells treated with RAD001.

### Integrins are modified in PC3^res^ cells

**Figure 3:**
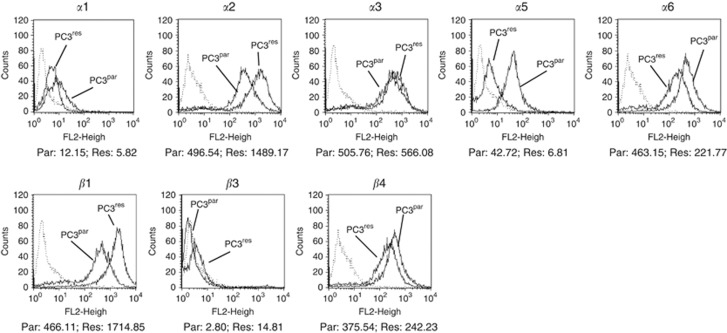
FACS analysis of integrin *α* and *β* subtype expression on PC3^par^ versus PC3^res^ cells. Cells were washed in blocking solution and then stained with specific monoclonal antibodies as listed in Materials and Methods. To evaluate background staining of PE-conjugated antibodies, goat anti-mouse IgG1-PE or IgG2a-PE was used (dotted lines). Fluorescence was analysed using a FACScan flow cytometer. Mean fluorescence values are given below the histograms. One from three independent experiments.

Surface levels of integrin *α* and *β* adhesion receptors were analysed next. The integrin subtypes *α*2, *α*3, *α*6, *β*1 and *β*4 were strongly expressed, *α*1 and *α*5 were moderately expressed and *β*3 was not expressed on PC3^par^ cells (Figure 3). The *α*4 integrin subtype was not detected by flow cytometry, either on PC3^par^ or PC3^res^ cells (data not shown). PC3^res^ cells were characterised by distinct differences in the integrin-expression pattern, compared with the controls. The *α*2 and *β*1 subtypes were dramatically elevated. Integrins *α*1 and *α*6 were lowered, whereas *α*5 was nearly lost on the PC3^res^ cell membrane. The *β*3 subtype appeared on PC3^res^ cells. Only slight differences were seen with respect to the *α*3 and *β*4 integrins. In a further experimental setting, PC3^res^ and PC3^par^ cells were treated short-term with 5 nℳ RAD001. Integrin *α*2 and *β*1 expression (most altered under chronic RAD001 treatment) was then evaluated. Exposing PC3^par^ cells to RAD001 led to an upregulation of *α*2 (+69.7±14.8%) and *β*1 (+39.7±7.6%), compared with untreated PC3^par^ cells. Short-term treatment of PC3^res^ cells with RAD001 also evoked an upregulation of *α*2 (+14.2±4.9%) and *β*1 (+17.3±4.0%), although to a lesser extent than in PC3^par^ cells.

**Figure 4:**
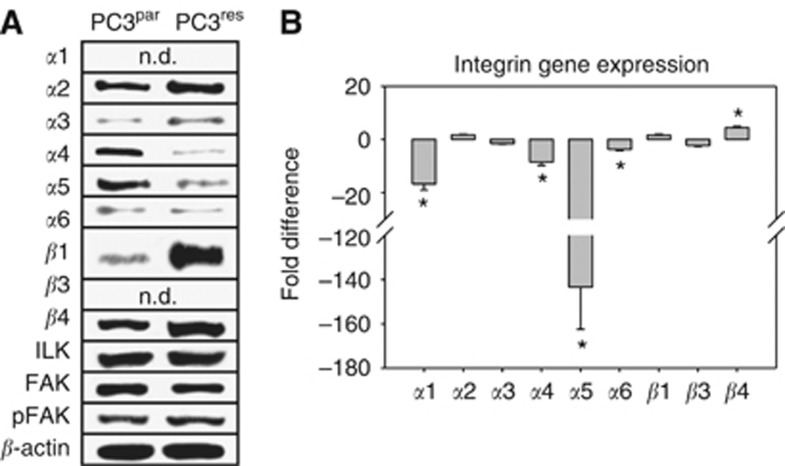
Modification of intracellular integrin protein level. (**A**) Lysates of PC3^par^ or PC3^res^ cells were subjected to SDS–PAGE and blotted on the membrane incubated with respective monoclonal antibodies. *β*-actin served as the internal control. The figure shows one representative from three separate experiments. (**B**) is related to the integrin gene-expression pattern. Primer sets used for evaluation are listed in materials and methods. Calculation of the relative expression of each gene was done by the ΔΔCt method in the analysis programme of SABioscience Corporation. The housekeeping gene GAPDH was used for normalisation. Values are given as fold difference to PC3^par^ cells. *indicates significant difference.

Western blotting demonstrated slight elevation of *α*2, *α*3 and *β*4 integrins in PC3^res^ cells, compared with the control cell line. Notably, the *β*1 protein content was found to be drastically upregulated in cells resistant to RAD001 (Figure 4A). In contrast, the *α*4 integrin protein, which was detectable in the PC3^par^ cytoplasm, as well as the *α*5 integrin, was suppressed in PC3^res^ cells. Protein bands specific for the *α*1 and *β*3 integrin were not seen in the cell cultures. FAK, pFAK and ILK analysis showed similar protein amounts in drug-resistant compared with drug-sensitive tumour cells.

Additionally, integrin-coding genes were evaluated. The most distinct differences became evident on *α*5 integrin mRNA, which was expressed in PC3^res^ at a very low level, compared with the PC3^par^ cells (Figure 4B). There was also a significant reduction of *α*1, *α*4 and *α*6 integrins, accompanied by an enhancement of *β*4 integrin mRNA in the resistant compared with non-resistant cells.

### Cell signalling is altered in PC3^res^ cells

**Figure 5:**
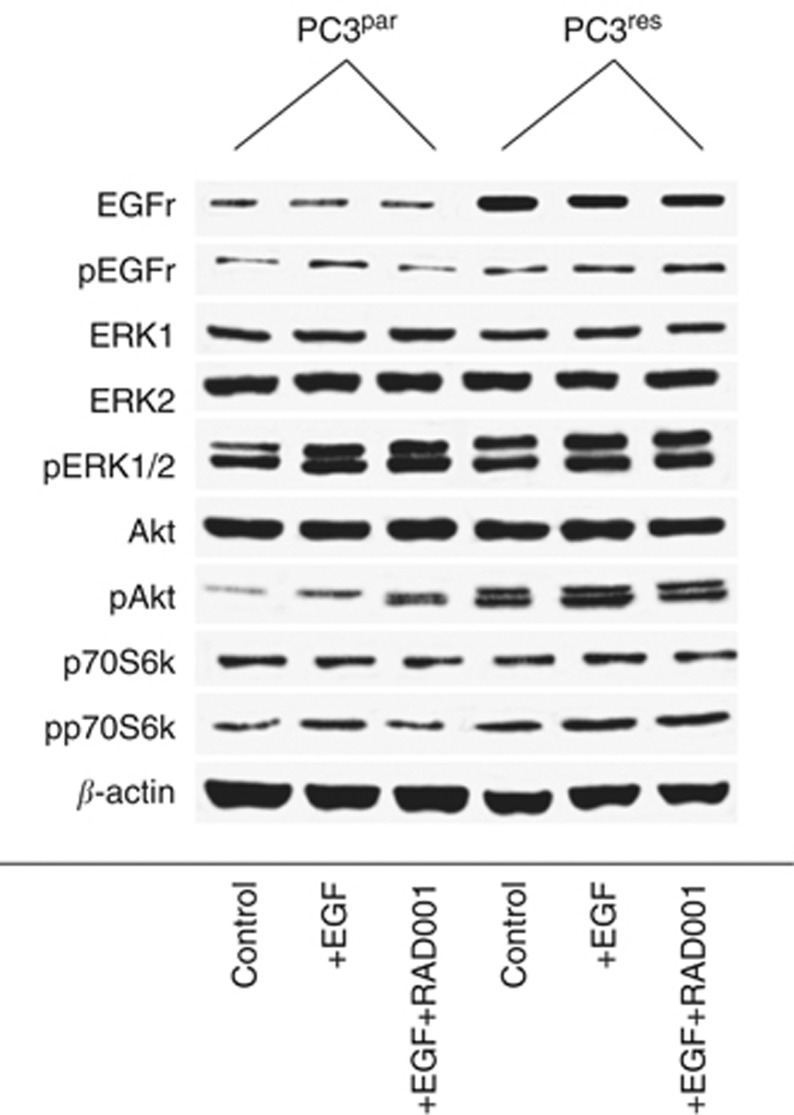
Western blot analysis of cell signalling proteins, listed in methods. PC3^par^ or PC3^res^ cells remained untreated (control). They were kept for 2 h in serum-free cell culture medium and subsequently stimulated for 30 min with EGF (100 ng ml^−1^; +EGF) or they were stimulated with EGF and additionally treated with 5 nℳ RAD001 (+EGF+RAD001). Cell lysates were then subjected to SDS–PAGE and blotted on the membrane incubated with the respective monoclonal antibodies. *β*-actin served as the internal control. The figure shows one representative from three separate experiments.

The consequence of resistance development for intracellular signalling was subsequently investigated. EGFr was expressed to a higher extent in PC3^res^ compared with PC3^par^ cells. This was also true with respect to EGFr activation (pEGFr; PC3^res^>PC3^par^). Stimulation with EGF further elevated pEGFr in both cell types. RAD001 reverted this process in PC3^par^ but not in PC3^res^ cells (Figure 5). p70S6k displayed a similar characteristic, whose activation in PC3^res^ exceeded that in PC3^par^ cells. RAD001 deactivated p70S6k in PC3^par^, whereas pp70S6k expression remained high in PC3^res^ cells. Akt was visualised as a faint band in PC3^par^ cells, whereas two distinct protein bands were detected in PC3^res^ cells. EGF elevated pAkt in both PC3^par^ and PC3^res^ cells. Addition of RAD001 further enhanced pAkt in PC3^par^ but slightly diminished pAkt in PC3^res^ cells. Phosphorylation of ERK was enhanced in PC3^res^ compared with PC3^par^ cells. EGF additionally enhanced pERK in both cell lines. However, RAD001 did not alter the activation status of this protein.

### Blocking studies

**Figure 6:**
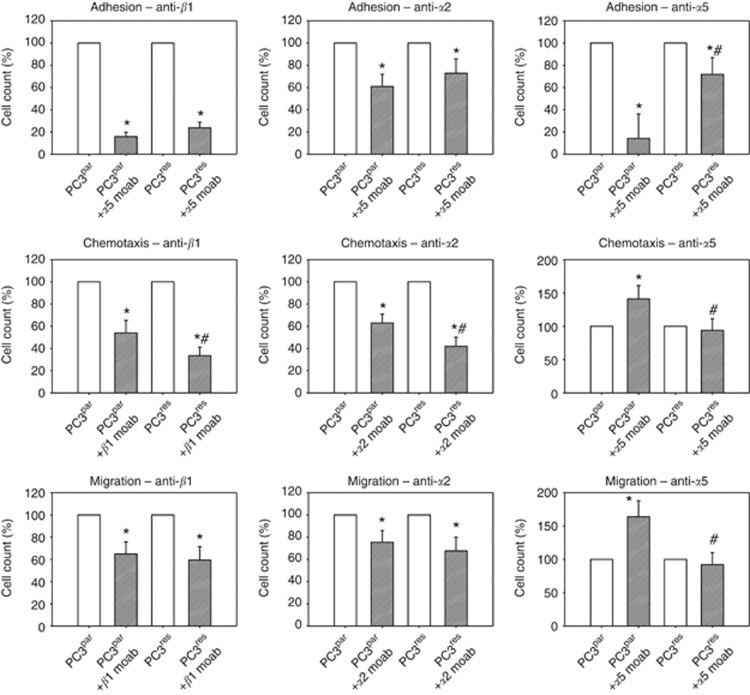
Influence of integrin *α*2, *α*5 or *β*1 blockade on tumour cell adhesion, chemotaxis or migration. PC3^par^ or PC3^res^ cells were preincubated for 60 min with function-blocking anti-integrin *β*1, anti-integrin *α*2 or anti-integrin *α*5 monoclonal antibodies. Controls remained untreated. Cells were then subjected to the adhesion, chemotaxis and migration assay as indicated in Materials and Methods. Values are shown as percentage difference to the 100% control. *indicates significant difference between the PC3 control subline and the PC3 subline treated with the function-blocking antibody. ^#^indicates significant difference between PC3^par^ and PC3^res^ cells whose integrin subtype was blocked.

To investigate the functionality of *β*1 and *α*2 integrins, which were strongly elevated in PC3^res^, compared with PC3^par^ cells, blocking studies were carried out. Figure 6 reveals that *β*1 and *α*2 integrins are significantly involved in adhesion, chemotactic movement and migration of both PC3^res^ and PC3^par^ cells. Chemotaxis was significantly more intensely diminished in PC3^res^ compared with PC3^par^ cells. We also investigated the relevance of the *α*5 integrin loss, which was evident in PC3^res^ cells. Blocking *α*5 in PC3^par^ cells led to a significant downregulation of adhesion and upregulation of chemotaxis and migration. However, blocking *α*5 in PC3^res^ caused no effect on the motile behaviour of this cell type. To explore whether activation of Akt, evident in the drug-resistant cells, is also involved in invasion and metastasis, PC3^par^ and PC3^res^ cells were treated with an Akt inhibitor and chemotaxis was investigated. Interestingly, Akt blockade strongly diminished chemotaxis of PC3^par^ (−43.9±10.2%) but not of PC3^res^ cells.

## Discussion

Despite encouraging preclinical and clinical results of mTOR inhibitors, resistance has emerged as a problem. Because metastasis is a critical step in tumour dissemination and progression, the consequences of RAD001 resistance in prostate cancer adhesion and invasion was investigated in the present study. The PC3^res^ cells were defined by an IC_50_ value for RAD001, which was 70-fold higher than that for PC3^par^ cells (Tsaur *et al*, 2011). Evidence is presented here that drug non-responsiveness is coupled to downregulation of tumour adhesion to endothelial cells and extracellular matrix proteins, accompanied by increased chemotactic activity. Tumour-cell amoeboid motility is necessary for metastasis (Yilmaz and Christofori, 2010; van Zijl *et al*, 2011). Hence, the differences seen between PC3^res^ and PC3^par^ cells indicate that long-term exposure to RAD001 alters intracellular mechanisms, which are closely involved in controlling metastatic spread. The differences in the motile behaviour of PC3^res^ and PC3^par^ cells became particularly evident in the chemotaxis assay, which only evaluates cell movement. Differences were not clearly seen in the migration and invasion assay, probably because these assays include cell movement as well as the interaction of the tumour cells with collagen or HUVEC, respectively. The interaction with HUVECs was downregulated in PC3^res^ compared with PC3^par^ cells. Consequently, the total count of PC3^res^ and PC3^par^ cells might be equalised in the migration and invasion assay. Most importantly, treating PC3^res^ cells with a therapeutically relevant RAD001 dosage dramatically increased their motile capability as shown in the chemotaxis, migration and invasion assays. Chronic drug treatment, therefore, may drive the tumour cell to acquire a more invasive phenotype, and continuing RAD001 application may further accelerate the metastatic dissemination.

Evaluation of the mechanism responsible for the elevated motile behaviour of PC3^res^ cells points to a modified integrin-expression pattern. Particularly, the *α*2 and *β*1 subtypes were upregulated, whereas the *α*5 subtype was absent in the drug-resistant cells. The role of *α*2 in prostate cancer metastasis is not yet clear. Neal *et al* (2011) have reported that *α*2 expression inversely correlates with prostate cancer cell migration into collagen, whereas the opposite was seen by Van Slambrouck *et al* (2009). Based on our own blocking studies, increased *α*2 seems more likely connected with elevated motile behaviour, because functional blocking of the integrin *α*2 subunit distinctly inhibited both chemotactic movement and migration through a collagen matrix. The blocking effect was significantly stronger in the resistant sublines than in the parental cells. This is important. Obviously, metastatic spreading of RAD001-resistant prostate cancer is accelerated by two strategies: (1) by upregulating the *α*2 expression level (quantitative regulation) and (2) by strengthening the relevance of *α*2 in controlling invasion (qualitative regulation). In fact, cell migration has been demonstrated to depend on the number of *α*2 integrin receptors expressed on the cell surface (Li *et al*, 2011), as well as on qualitative parameters, such as activation of intracellular signalling cascades and/or receptor cross-talk (Ning *et al*, 2005; Sawhney *et al*, 2006). We assume that the conversion of prostate cancer cells from a drug-sensitive to a drug-insensitive state is accompanied by an elevated *α*2 level, *α*2–cytoskeleton interaction and cytoskeleton-related signalling, finally enforcing actin turnover and remodelling.

The same mode of action may be attributed to *β*1 as to *α*2 integrin receptors, because *β*1 blockade leads to a distinct downregulation of chemotaxis and migration (PC3^res^>PC3^par^). However, the role of the *β*1 receptor seems to be complex. Blocking *β*1 also reduced tumour cell adhesion properties. Because *β*1 was strongly increased in the PC3^res^ variant, an enhanced attachment rate of these cells, compared with the controls, could be expected, but was not the case. PC3^res^–HUVEC and PC3^res^–matrix interaction were even lowered. Consequently, integrin *β*1 may not serve as a pure mechanistic binding element. Live cell imaging of fibroblast spreading has demonstrated that *β*1 undergoes an affinity switch, which allows disassembly of adhesion structures and dynamic crawling (Millon-Frémillon *et al*, 2008). In line with this, alteration of *β*1-actin cross-linking has been reported to weaken adhesion and increase migratory activity of cancer cells (Mouneimne and Brugge, 2007). Chronic treatment with RAD001 possibly induces a functional switch in PC3 cells. In fact, a greater dependency of tumour migration on *β*1 was seen in the resistant compared with the non-resistant cell line. With this in mind, *β*1 (as well as *α*2) integrin elevation in PC3^par^ induced by short-term RAD001 application may strengthen adhesive forces and thereby prevent motile spreading, whereas the same effect may cause enhanced chemotactic activity of PC3^res^ cells. In fact, treating PC3^par^ cells with RAD001 led to a significant reduction of tumour cell chemotaxis, migration and invasion, whereas short-term treatment of PC3^res^ cells with RAD001 evoked the opposite effect. The interpretation of the integrin data obtained after short-term RAD001 treatment is speculative. However, the same integrin has recently been shown to control cell spreading and retraction by switching the direction of integrin outside-in signalling (Flevaris *et al*, 2007). Deshmukh *et al* (2011) have provided a complex paradigm where integrin function depends on the secondary structure pattern and overall folding of the integrin cytoplasmic tail, shifting the integrin influence to different signalling proteins and the intracellular pathways. Therefore, it seems plausible that resistance development of PC3 cells may be accompanied by two different processes: (A) quantitative alterations of the integrin-expression level and (B) structural changes of the integrin molecules, leading to a switch of the intracellular pathway direction following short-term RAD001 treatment.

Apart from being involved in metastasis, *β*1 integrins are required for Akt phosphorylation and contribute to cell survival and growth (Riaz *et al*, 2012). Downregulation of *β*1 in prostate cancer cells inhibited Akt activation and retarded tumour proliferation (Niewiarowska *et al*, 2009; Goel *et al*, 2010). Meanwhile, *β*1 is considered to be a key component in regulating the conversion from a dormant state to active proliferation and metastasis (Barkan and Chambers, 2011). Our data point to a strong activation of Akt (along with EGFr and pERK) in PC3^res^ cells. We cannot definitively declare that the massive accumulation of *β*1 in PC3^res^ cells activates growth-related signals, because we did not analyse *β*1–Akt cross communication. However, Akt activation points towards a speed up of the cell-cycle machinery. It is of particular interest that *β*1 has been shown to contribute to chemoresistance in head and neck (Eke *et al*, 2012), pancreatic (Danilov *et al*, 2011), breast (Huang *et al*, 2011), lung (Ju *et al*, 2010) and ovarian cancer (Chen *et al*, 2010a), and targeting β1 integrins has provided benefit in overcoming drug non-responsiveness (Mori *et al*, 2008; Park *et al*, 2008; Huang *et al*, 2011). Whatever the precise mechanism of *β*1 in PC3^res^ is, it represents a significant prognostic and therapeutic marker molecule. From a clinical viewpoint, patients should be carefully controlled when a *β*1 increase becomes overt. Ongoing studies should explore whether *β*1 increases during chronic RAD001 treatment and whether this increase correlates with resistance development in cancer patients.

An interesting phenomenon is seen with respect to the *α*5 integrin. Blocking *α*5 led to decreased adhesion and increased chemotaxis and migration of PC3^par^ cells. It has recently been postulated that *α*5 may be crucial for cell detachment and subsequent metastasis of prostate cancer (Neal *et al*, 2011), which is in line with our results. However, this relationship does not seem transferable to the PC3^res^ cells, whose adhesion properties were only slightly, and motile behaviour not at all, modified following *α*5 blockade. Another mode of action must be assumed here. Experiments with breast (Wang *et al*, 2011), melanoma (Landreville *et al*, 2011) or colon cancer cells (De Wever *et al*, 2011) have shown that α5 subunit functions as a tumour-growth suppressor. Indeed, a link between the *α*5 integrin and cell-cycle controlling proteins exists, because overexpression of *α*5 triggers downregulation of CDK2, thereby inhibiting cellular entry into the S phase (Wang *et al*, 2011). Vice versa, loss of *α*5 as seen in the PC3^res^ cells may trigger enhanced CDK2 expression, resulting in elevated mitotic activity. This is speculative. However, a recent publication points to the accumulation of CDK1 and CDK2 in RAD001-resistant prostate cancer cells (Tsaur *et al*, 2011), which supports our hypothesis that the reduction of *α*5 evoked by long-term RAD001 exposure may cause an increase in tumour growth.

With respect to intracellular signalling, the most striking differences were seen in the activation level of Akt, which was strongly enhanced in PC3^res^ compared with PC3^par^ cells. Much data point to the relevance of this protein in resistance development. Upregulation of phosphorylated Akt has been shown to correlate to docetaxel resistance and progression to castration-resistant prostate cancer after androgen ablation (Kosaka *et al*, 2011). Evidence has also been provided that the Akt pathway has an important role in TRAIL resistance in cancer cells (Xu *et al*, 2010). It is not clear how long-term inhibition of mTOR triggers Akt activation. mTOR consists of two complexes, mTORC1, which is located downstream of Akt and is sensitive to mTOR inhibitors, and mTORC2, which is upstream of Akt and is resistant to mTOR inhibitors (Ma and Blenis, 2009). Long-term application of RAD001 may, therefore, induce feedback activation of Akt via mTORC2 signalling.

Concerning metastatic progression, activation of the Akt pathway has been shown to correlate with the chemotactic motility of prostate cancer cells *in vitro* (Jeong *et al*, 2012) and prostate tumour progression to metastasis in the transgenic adenocarcinoma mouse prostate mouse model (Sakamoto *et al*, 2010). Indeed, Akt blockade strongly diminished chemotaxis of PC3^par^ cells, which corroborates both reports. Surprisingly, chemotactic activity of PC3^res^ cells was not diminished following Akt blockade, perhaps indicating uncoupling of the integrin–Akt axis during resistance development. Similarly, Chen *et al* (2010b), recently observed an uncoupling of the Akt-connected pathways in drug-resistant breast cancer cells. This finding could be clinically relevant because therapeutic suppression of Akt may no longer prevent metastatic progression once tumour cells have acquired resistance. Whether the action of Akt in PC3^res^ cells is exclusively focused on increasing the tumour mass (e.g., by speeding up tumour cell proliferation and blocking apoptosis) is not yet clear.

This study demonstrates that RAD001 resistance drives prostate cancer cells to become highly motile. The process is accompanied by significant alterations of the integrin-expression profile, particularly *α*2, *α*5 and *α*1, and by reactivating Akt. Further studies should be directed towards answering whether *α*5 integrin undergoes a functional switch from adhesion/migration to proliferation under chronic RAD001 treatment and whether Akt is connected to integrins during resistance development.
